# A 41-Year-Old Woman with Seizure

**Published:** 2017-04-15

**Authors:** Fatemeh Mohammadi, Reza Mosaddegh, Samira Vaziri

**Affiliations:** 1 Emergency Medicine Management Research Center, Iran University of Medical Sciences, Tehran, Iran.

## Case presentation:

The patient was a 41 year old woman that was brought to the emergency department (ED) by her husband following seizure. According to the relatives accompanying her, the seizure was of tonic-clonic type, had occurred one hour before presentation to ED, and had lasted 3 minutes. The patient had been drowsy for about 15 minutes after the end of the seizure. She had no history of head trauma and did not mention headache, nausea and vomiting, fever, vision problems or others. On presentation, amnesia regarding the things that happened was evident. In her history, she had a generalized tonic-clonic seizure 4 years back, regarding which she had not done proper follow up for taking necessary diagnostic measures and had not been treated with anti-epileptic medication. She had a history of surgery for removing cold thyroid nodule 20 years ago and was under calcium treatment for 15 years but she had decided to stop taking her medications since 5 year ago. She did not have a history of alcohol or drug abuse. The patient was conscious and awake on presentation and did not have any specific clinical complaints. Her vital signs on presentation were as follows:

Blood Pressure = 120/70mmHg, Pulse Rate = 68/minute, Respiratory Rate = 16/minute, O2saturation= 98% at air room, Oral Temperature= 37°C, and bedside blood sugar in the normal range.

**Figure 1:**
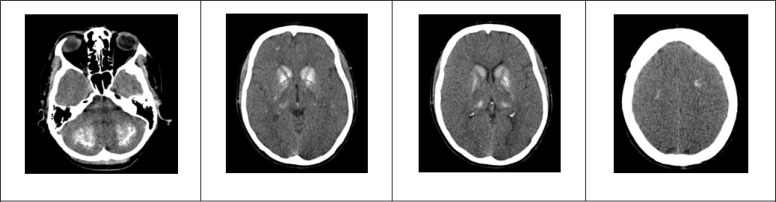
Patient’s axial cuts of brain computed tomography scan without contrast materials.

In head and neck examination, the surgery scar was seen in the thyroid region. Examination of the thyroid also showed a nodular surface in palpation. Examination of the heart, lungs, abdomen and extremities did not have pathologic findings. Neurologic examinations including evaluation of cranial nerves, sense and power of the muscles, cerebellar examination and deep tendon reflexes were normal. Based on the opinion of the in-charge physician, anti-epileptic drugs were not prescribed at this stage. Laboratory tests including complete blood cell count, liver function tests (LFT), and level of blood sugar (BS), sodium, potassium, calcium, phosphorus, magnesium, urea and creatinine electrolytes were ordered. Considering the full consciousness of the patient and stability of vital signs and clinical examinations, by taking safety measures and accompanied by a nurse, the patient was referred to the radiology unit to undergo a brain computed tomography (CT) scan, the results of which are shown in figure 1.

**What is your diagnosis?**

**Diagnosis:**

In the patient’s CT scan, numerous calcifications were detected in the sub-cortical region, cerebellum, and basal ganglia. Results of the laboratory tests of magnesium, potassium, sodium, creatinine, urea, LFT, and BS were in the normal range. However, calcium = 5.9 mg/dl and phosphorus = 7.8 mg/d were reported. Serum level of parathyroid hormone (PTH) was measured and PTH = 5.2 pg/mL was reported. 

Considering the history of thyroid surgery and brain calcinosis in CT scan images as well as hypocalcemia, hyperphosphatemia, and low serum level of parathyroid hormone in the tests, iatrogenic hypoparathyroidism was confirmed.

**Case fate:**

Treatment started with prescription of calcium and the patient received consultation with neurology and endocrinology services. In thyroid ultrasonography, a hyperechoic nodule that measured 13×14 with coarse calcification was seen in the right lobe of the thyroid and a 15×20 nodule was found in the left lobe of the thyroid. During the hospitalization the patient did not have another seizure and was finally discharged with good general condition and hypopatathyroidism diagnosis. She underwent outpatient follow up and was also put in line for thyroidectomy surgery.

## Discussion

Hypoparathyroidism is an endocrine disorder that occurs due to various reasons including genetic, autoimmune, idiopathic, or iatrogenic causes (1). Damage to parathyroid glands following surgery in the neck area and particularly thyroidectomy, is the most common etiology detected for this problem that despite taking all the precautious measures and proper skill of the surgeon happens temporarily in 20-30% of the cases and permanently in 1-7% (2). Hypoparathyroidism after thyroid surgery happens due to direct damage to the parathyroid glands or as a result of damage to its blood supplying vessels (3, 4).

Neuromuscular symptoms are the most common clinical manifestations of hypoparathyroidism. Patients usually present with complaints of paresthesia, muscle cramps, tetany, and carpopedal spasm. However, it may also manifest as seizure, neurocognitive disorder, bronchospasm, laryngospasm, or cardiac rhythm disorders (3, 5). Chronic hypocalcemia due to hypoparathyroidism caused by surgery can be subclinical for long periods of time (6). Seizure and psychological disorders in this disease occur due to hypocalcemia and are reversible after correction of calcium. Cognitive and extrapyramidal disorders are seen due to advanced calcification of brain tissue, usually in late diagnosis cases. Manifestation of hypocalcemia symptoms in patients who undergo total thyroidectomy occurs more rapidly compared to those who undergo sub-total thyroidectomy and due to removal of more thyroid tissue and more damage to parathyroid glands, the drop in calcium is also more rapid (7).

Hyperphosphatemia caused by hypoparathyroidism can lead to ectopic calcification in kidney, eye, vessels, and brain in the long run (2). Observation of calcinosis in brain imaging of a patient with a history of thyroidectomy should make the physician suspicious of hypoparathyroidism and guide them towards doing more evaluations (6). However, calcification of basal ganglion has numerous causes; the most common of which is calcium and phosphorus imbalance, yet some people develop this problem without an apparent cause, which is called Fahr syndrome (8). Initially, serum levels of calcium, phosphorus, and parathormone hormone should be measured. If hypocalcemia, hyperphosphatemia and low level of parathormone are detected, it is necessary to measure serum levels of ionized calcium and magnesium in the next step. If serum magnesium level is normal and serum level of ionized calcium is low, the diagnosis is confirmed (9). Hypomagnesiumia is one of the rarest and reversible causes of hypothyroidism that has a completely different treatment compared to post-surgery hypoparathyroidism (2). 

The treatment for this problem in ED is prescription of calcium. For this purpose it has been recommended to use calcium gluconate and avoid injection of calcium chloride due to potentially sclerosing to veins (9). Anti-epileptic drugs alone are not effective in controlling the seizure caused by electrolyte imbalances and rapid identification and correction of these imbalances, if present, is necessary for controlling seizure and preventing brain disorders (10). About 54% of the patients need anti-epileptic drugs for controlling seizure. Phenytoin is epileptogenic in presence of hypocalcemia, since it induces liver enzymes and increases metabolism of vitamin D to its inactive form. Therefore, it is better to use other anti-epileptic drugs in these patients if needed (5, 11).

When encountering patients with seizure presenting to ED, taking accurate history and especially asking about their history of thyroid surgery can lead to clinical suspicion to hypoparathyroidism. Confirming or ruling out this diagnosis can be easily and rapidly done via measuring serum levels of calcium and phosphorus. By detecting hypocalcemia and hyperphosphatemia, instead of unnecessary prescription of anti-epileptic drugs, proper treatment can be done.

## References

[1] Agarwal R, Lahiri D, Biswas A, Mukhopadhyay J, Maity P, Roy M (2014). A rare cause of seizures, parkinsonian, and cerebellar signs: brain calcinosis secondary to thyroidectomy. North American journal of medical sciences.

[2] Shoback DM, Bilezikian JP, Costa AG, Dempster D, Dralle H, Khan AA (2016). Presentation of hypoparathyroidism: etiologies and clinical features. The Journal of Clinical Endocrinology & Metabolism.

[3] Kakava K, Tournis S, Papadakis G, Karelas I, Stampouloglou P, Kassi E (2015). Postsurgical Hypoparathyroidism: A Systematic Review. In vivo (Athens, Greece).

[4] Zhou HY, He JC, McHenry CR (2016). Inadvertent parathyroidectomy: incidence, risk factors, and outcomes. Journal of Surgical Research.

[5] Bhadada S, Bhansali A, Upreti V, Subbiah S, Khandelwal N (2010). Spectrum of neurological manifestations of idiopathic hypoparathyroidism and pseudohypoparathyroidism. Neurology India.

[6] Zisimopoulou V, Siatouni A, Tsoukalos G, Tavernarakis A, Gatzonis S (2012). Extensive bilateral intracranial calcifications: a case of iatrogenic hypoparathyroidism. Case reports in medicine.

[7] Hosseini M, Otaghvar H, Tizmaghz A, Shabestanipour G, Vahid P (2016). Evaluating the Time Interval for Presenting the Signs of Hypocalcaemia after Thyroidectomy. Journal of clinical and diagnostic research: JCDR.

[8] Rossi M, Morena M, Zanardi M (1993). Calcification of the basal ganglia and Fahr disease. Report of two clinical cases and review of the literature. Recenti progressi in medicina.

[9] Bilezikian JP, Khan A, Potts JT, Brandi ML, Clarke BL, Shoback D (2011). Hypoparathyroidism in the adult: Epidemiology, diagnosis, pathophysiology, target‐organ involvement, treatment, and challenges for future research. Journal of Bone and Mineral Research.

[10] Nardone R, Brigo F, Trinka E (2016). Acute Symptomatic Seizures Caused by Electrolyte Disturbances. Journal of clinical neurology (Seoul, Korea).

[11] El Otmani H, Lahlou I, Raji L, Omari S, Belmansour Y, Moutaouakil F (2012). Striatopallidodentate calcinosis, hypoparathyroidism and neurological features: a case series study. Revue neurologique.

